# Castleman Disease Presenting as Unexplained Abdominal Pain in a 93-Year-Old Woman: A Case Report

**DOI:** 10.7759/cureus.72844

**Published:** 2024-11-01

**Authors:** Chihiro Uda, Ryuichi Ohta, Yoko Senaha, Chiaki Sano

**Affiliations:** 1 Family Medicine, Fuchu Hospital, Osaka, JPN; 2 Community Care, Unnan City Hospital, Unnan, JPN; 3 Digestive and General Surgery, Shimane University Faculty of Medicine, Izumo, JPN; 4 Community Medicine Management, Shimane University Faculty of Medicine, Izumo, JPN

**Keywords:** abdominal pain, castleman disease, corticosteroid therapy, lymphadenopathy, lymphoproliferative disorders, unicentric castleman disease

## Abstract

Castleman disease (CD) is a rare lymphoproliferative disorder that can present with nonspecific symptoms, making diagnosis challenging, particularly in elderly patients. This case report describes a 93-year-old woman who presented with intermittent abdominal pain, which was unresponsive to standard treatments and showed no systemic signs of infection or malignancy. Initial imaging revealed scattered lymphadenopathy, raising concern for a lymphoproliferative disorder. A laparoscopic lymph node biopsy was performed, leading to a diagnosis of unicentric CD (UCD). The patient was treated with corticosteroids, which resulted in a marked improvement in her symptoms, including the complete resolution of her abdominal pain. This case emphasizes the importance of considering CD in the differential diagnosis of unexplained abdominal pain in elderly patients, as early diagnosis and treatment can prevent complications and significantly improve outcomes. The successful use of corticosteroids in this patient highlights a nonsurgical treatment option for UCD in cases where surgical excision is not feasible.

## Introduction

Castleman disease (CD), first reported by pathologist Benjamin Castleman in 1956 at Massachusetts General Hospital, is a rare lymphoproliferative disorder [1]. The initial case involved a mediastinal mass resembling thymoma, with histological features characterized by marked lymphoid follicular hyperplasia, vascular endothelial proliferation, and prominent angiogenesis [2]. Subsequently, another research described another subtype where numerous plasma cells were found in the interfollicular spaces [3]. Based on these histological differences, CD is categorized into three types: hyaline vascular type, plasma cell type, and mixed type, which combines features of both.

The annual incidence of CD in the United States is estimated at approximately 2,000 cases as of a 2022 study. In a 2014 study, about 75% of cases were classified as unicentric CD (UCD), while the remaining 25% were multicentric CD (MCD), further subdivided into human herpesvirus 8 (HHV8)-associated MCD and idiopathic (HHV8-negative) MCD [2]. A 2022 study also estimated that approximately 40% of CD cases involve UCD [4].

CD is known to present with a variety of nonspecific symptoms and can affect individuals of all ages. As a benign lymphoproliferative disorder, its diagnosis requires careful exclusion of other conditions, such as chronic infections, autoimmune diseases, and malignancies, which may present with similar symptoms and histological findings [5,6]. In this case report, we present a 93-year-old woman diagnosed with CD during an evaluation for unexplained abdominal pain. This case highlights the importance of a thorough diagnostic workup in elderly patients with nonspecific symptoms, leading to the timely identification of treatable conditions such as CD.

## Case presentation

A 93-year-old woman presented to a community hospital with a complaint of intermittent abdominal pain, localized from the epigastrium to the lower abdomen, persisting for several weeks. The pain was described as stabbing and lasted approximately 30 minutes at a time, with no identifiable triggers, such as food intake or body movements. Although the intensity of the pain remained unchanged, its frequency had increased, prompting her to seek medical attention. The patient denied experiencing fever, headache, chest pain, dyspnea, back pain, night sweats, nausea, vomiting, or diarrhea. She had no history of contact with infected individuals or recent travel. Her medical history included hypertension, dyslipidemia, chronic heart failure, and gastroesophageal reflux disease. She was on a regimen of valsartan (80 mg), amlodipine (2.5 mg), carvedilol (10 mg), pravastatin (10 mg), and esomeprazole (10 mg).

On presentation, her vital signs were as follows: body temperature: 37.1 °C, blood pressure: 143/87 mmHg, heart rate: 83 beats per minute, SpO2: 96% on room air, and respiratory rate: 14 breaths per minute. Physical examination revealed tenderness upon palpation in the epigastric and lower abdominal regions without guarding or rebound tenderness. No skin rash or other abnormal findings were observed. The laboratory test showed hyperinflammatory conditions such as increased CRP, erythrocyte sedimentation rate, and IgG (Table 1).

**Table 1:** Initial laboratory data of the patient

Parameter	Level	Reference
White blood cells	5.8	3.5–9.1 × 10^3^/μL
Neutrophils	77.8	44.0–72.0%
Lymphocytes	13.8	18.0–59.0%
Hemoglobin	10.9	11.3–15.2 g/dL
Hematocrit	31.7	33.4–44.9%
Mean corpuscular volume	85.7	79.0–100.0 fl
Platelets	12.4	13.0–36.9 × 10^4^/μL
Erythrocyte sedimentation rate	58	2–10 mm/hour
Total protein	7.3	6.5–8.3 g/dL
Albumin	2.9	3.8–5.3 g/dL
Total bilirubin	0.7	0.2–1.2 mg/dL
Aspartate aminotransferase	58	8–38 IU/L
Alanine aminotransferase	21	4–43 IU/L
Lactate dehydrogenase	415	121–245 U/L
Blood urea nitrogen	13.5	8–20 mg/dL
Creatinine	0.79	0.40–1.10 mg/dL
Serum Na	140	135–150 mEq/L
Serum K	3.5	3.5–5.3 mEq/L
Serum Cl	106	98–110 mEq/L
Ferritin	1613.4	14.4–303.7 ng/mL
CRP	2.82	<0.30 mg/dL
IgG	1861	870–1700 mg/dL
IgM	179	35–220 mg/dL
IgA	681	110–410 mg/dL
Urine test	-	-
Leukocyte	Negative	Negative
Protein	Negative	Negative
Blood	Negative	Negative

**Figure 1:**
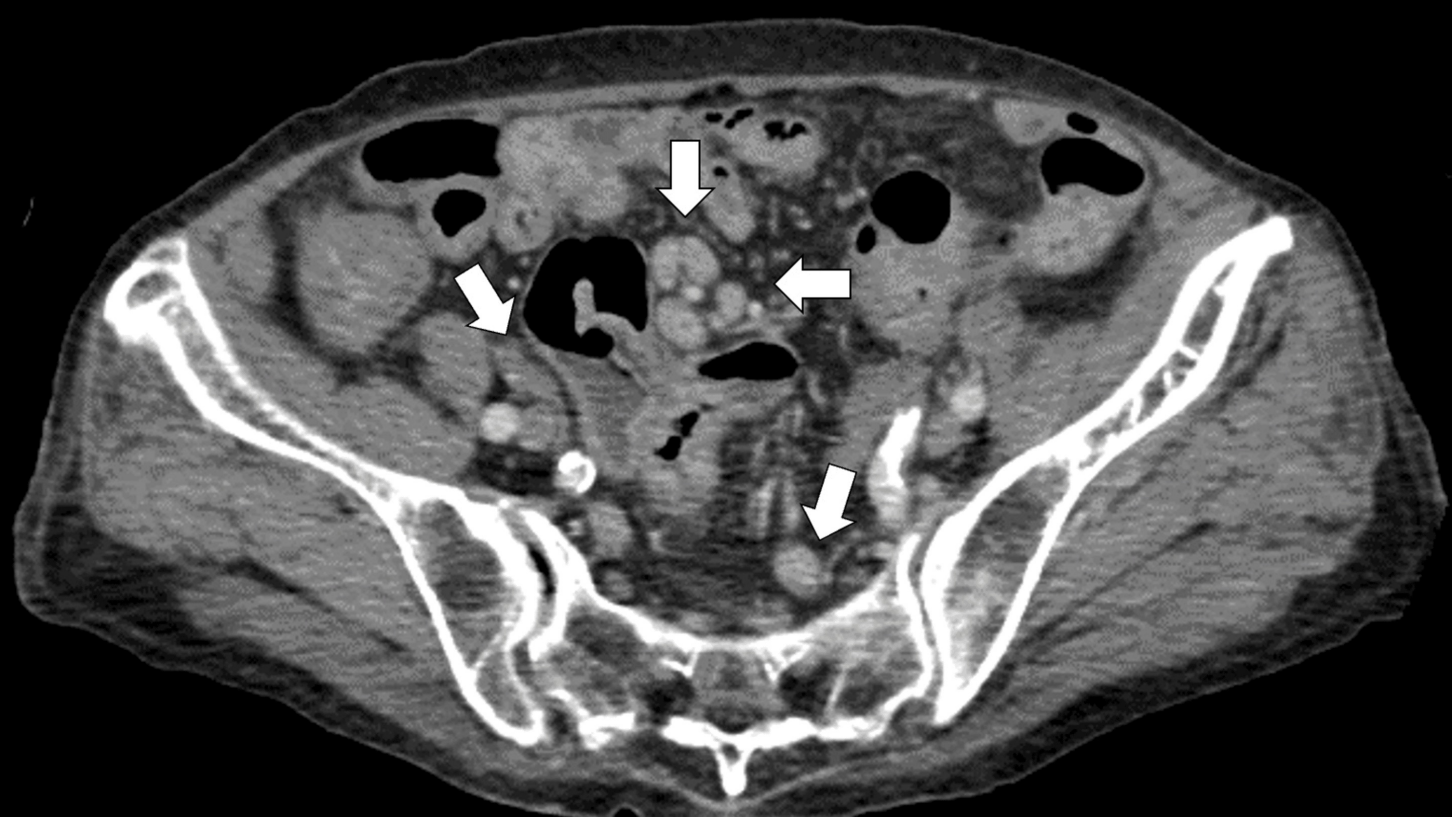
Enhanced CT demonstrating scattered lymphadenopathy around the aorta and peritoneum, raising concern for carcinomatosis (white arrows)

Abdominal ultrasound showed a small amount of free fluid in Douglas’s pouch, prompting further investigation with a CT scan. The CT scan demonstrated scattered lymphadenopathy around the aorta and peritoneum, raising concern for carcinomatosis (Figure 1).

The patient was admitted for further evaluation, including upper and lower gastrointestinal endoscopy, both unremarkable. Given the possibility of a lymphoproliferative disorder, the patient was referred to the surgical department for a lymph node biopsy. Although the patient and her family initially declined further invasive procedures, they agreed to proceed with the biopsy after a thorough explanation of the potential diagnosis and treatment options. On the 15th hospital day, the patient underwent laparoscopic-assisted lymph node biopsy, which revealed no overt malignant findings intraoperatively, but scattered lymphadenopathy was observed.

**Figure 2:**
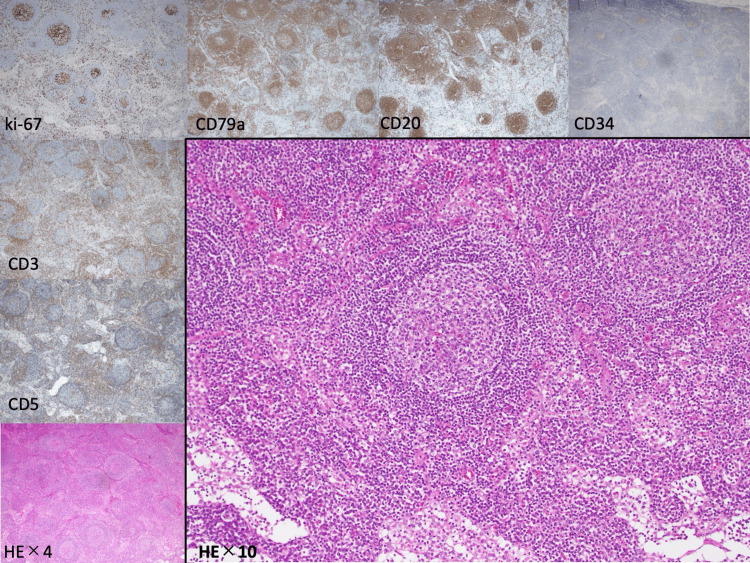
Histopathological examination of the biopsied lymph node Lymphoid follicular hyperplasia and proliferation of lymphocytes were demonstrated by immunohistochemistry, showing no significant imbalance between B cells (CD79a, CD20) and T cells (CD3, CD5) without the positivity of CD34.

Histopathological examination of the biopsied lymph nodes showed lymphoid follicular hyperplasia and proliferation of lymphocytes. Immunohistochemistry demonstrated no significant imbalance between B cells (Classification of differentiation (CD) 79a, CD20) and T cells (CD3, CD5) without the positivity of CD34, leading to the diagnosis of CD (Figure 2).

Following the initiation of corticosteroid therapy with 15 mg of prednisolone per day, the patient’s abdominal pain gradually resolved, and her appetite improved. Following this improvement, the patient was discharged in good condition.

## Discussion

This case report provides an important example of how CD, a rare lymphoproliferative disorder, can present as unexplained abdominal pain in elderly patients, demonstrating the necessity of considering this diagnosis even in cases with nonspecific clinical manifestations [7]. CD can mimic various other conditions, including malignancies and chronic inflammatory diseases, making its diagnosis particularly challenging, especially in older adults with multiple comorbidities [4,8]. In this patient, the diagnosis was only confirmed following a lymph node biopsy, which underscores the critical role of tissue pathology in diagnosing lymphoproliferative disorders.

CD can manifest in two primary clinical subtypes: UCD and MCD. UCD generally affects a single lymph node region, whereas MCD involves multiple lymph node areas and often presents with systemic symptoms such as fever, night sweats, and weight loss [9]. In the present case, the patient’s intermittent abdominal pain was associated with localized lymphadenopathy without systemic manifestations, which is less common among UCD [10]. This localized presentation, alongside the absence of infectious or malignant causes, should raise the suspicion of a benign lymphoproliferative disorder, ultimately leading to the diagnosis of UCD.

Despite being a benign condition, UCD can still have significant clinical implications. Left untreated, lymphadenopathy may progress, leading to complications such as compression of adjacent organs or even transformation into more aggressive disorders like lymphoma or follicular dendritic cell sarcoma [11]. Thus, early diagnosis and management are critical for improving outcomes, especially in elderly patients, where maintaining quality of life is paramount. In this case, after diagnosis, the patient responded well to corticosteroid therapy aimed at reducing inflammation, with complete resolution of abdominal pain, suggesting that early intervention can be highly effective in symptom control.

It is also noteworthy that CD is associated with significant heterogeneity in its presentation and progression. While the majority of UCD cases remain stable over time and respond well to surgical excision, some patients may develop paraneoplastic syndromes or secondary malignancies, underscoring the importance of ongoing surveillance. The annual incidence of CD in the United States is estimated at approximately 2,000 cases, with UCD comprising about 40%. While the incidence rates between the United States and Japan are thought to be similar, MCD is more frequently observed in Japan, and HHV8-related MCD is relatively rare [12]. This highlights potential geographical and epidemiological variations in CD presentations, which may affect diagnostic and treatment approaches.

In elderly patients with unexplained abdominal pain, especially those with comorbidities that complicate the clinical picture, considering lymphoproliferative disorders like CD is crucial. Imaging studies are often nonspecific, as seen in this case, where scattered lymphadenopathy was detected on a CT scan [13]. This makes histopathological examination essential for a definitive diagnosis. Furthermore, this case underscores the importance of collaboration between general practitioners and specialists in the management of complex, rare diseases [14]. The decision to perform a lymph node biopsy in this case, despite initial reservations due to the patient’s age and the absence of overt systemic illness, was pivotal in achieving the correct diagnosis and subsequent treatment.

From a therapeutic perspective, UCD is generally curable with complete surgical resection of the affected lymph nodes, considered the gold standard treatment. However, when surgery is not feasible, corticosteroids or other immunosuppressive therapies can be considered, as demonstrated in this case [15]. Recognizing that UCD has a favorable prognosis with appropriate management is essential, and timely intervention can prevent complications and improve patient outcomes.

Finally, the case highlights the need for a high index of suspicion for CD in older adults, mainly when the clinical presentation involves unexplained abdominal pain and lymphadenopathy. Early diagnosis and treatment are essential to avoid unnecessary delays in care and prevent progression to more severe disease manifestations.

## Conclusions

This case of a 93-year-old woman diagnosed with CD following investigations for unexplained abdominal pain emphasizes the importance of including lymphoproliferative disorders in the differential diagnosis of elderly patients with nonspecific symptoms. Imaging studies, followed by biopsy, were critical in establishing the diagnosis, and subsequent corticosteroid treatment led to a favorable clinical outcome. This case underscores the need for a thorough diagnostic approach, even in elderly patients, to ensure appropriate and timely treatment for reversible conditions like CD.
